# Reliability, diagnostic value, and diagnostic yield of ultrasound‐guided percutaneous core needle biopsy for peritoneal lesions

**DOI:** 10.1002/cam4.7467

**Published:** 2024-07-17

**Authors:** Muhammet Arslan, Halil Serdar Aslan, Ayse Ruksan Utebey, Sercan Vurgun, Erdem Comut, Serkan Degirmencioglu, Derya Kılıc

**Affiliations:** ^1^ Department of Radiology Pamukkale University Faculty of Medicine Denizli Turkey; ^2^ Department of Radiology Tavas State Hospital Denizli Turkey; ^3^ Department of Pathology Pamukkale University Faculty of Medicine Denizli Turkey; ^4^ Department of Oncology Pamukkale University Faculty of Medicine Denizli Turkey; ^5^ Department of Gynecology and Obstetrics Pamukkale University Faculty of Medicine Denizli Turkey

**Keywords:** core needle biopsy, omental masses, percutaneous biopsy, peritoneal lesion, ultrasonography, ultrasound‐guided biopsy

## Abstract

**Introduction:**

Peritoneal lesions cannot be definitively distinguished based on clinical and imaging characteristics alone. This study aimed to evaluate the reliability, diagnostic value, and diagnostic yield of ultrasound‐guided percutaneous core needle biopsy (PCNB) for peritoneal lesions.

**Methods:**

A retrospective analysis of 129 patients who underwent PCNB for peritoneal lesions was performed to assessed technical completion and diagnostic yield.

**Results:**

The results showed that ultrasound‐guided PCNB is a safe and reliable diagnostic tool with high diagnostic yield for peritoneal lesions. Technical feasibility and diagnostic yield rates were 100% and 89.9%, respectively. The diagnostic yield was lower for patients with a known history of cancer and a short anteroposterior diameter of the target lesion.

**Conclusions:**

These findings suggest that ultrasound‐guided PCNB could be considered as a first‐line diagnostic tool for peritoneal lesions, as it offers a minimally invasive and accurate means of obtaining tissue samples for diagnosis.

## Graphical abstract

Percutaneous ultrasound‐guided needle core biopsy is a safe and reliable diagnostic tool with high diagnostic yield for peritoneal lesions. History of cancer and the anteroposterior diameter of the target lesion affect the diagnostic yield of percutaneous biopsy.

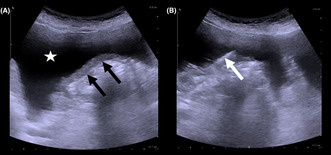

## 1 INTRODUCTION

Peritoneal pathologies are easily visible on radiological examinations but are often insufficient for identifying the etiology.^1^ Additionally, imaging findings may inconclusive, and a correct diagnosis may not be possible with diagnostic imaging. As peritoneal fluid is not always present and fluid cytology has a low diagnostic value, imaging‐guided core biopsy remains extremely important.^2^ Therefore, tissue diagnosis with a biopsy is frequently required and important for treatment. Open surgery and laparoscopy are effective techniques, but these are not the first choice because they are more invasive than percutaneous biopsies for tissue diagnosis. Ultrasound‐guided percutaneous core needle biopsy (PCNB) is a useful method for histologic diagnosis of peritoneal lesions.^3^ Ultrasound‐guided percutaneous biopsy of peritoneal lesions can be technically challenging because of the localization of the lesions and their proximity to the gastrointestinal tract, solid organs, or vessels. The factors influencing the diagnostic yield of PCNB have not been sufficiently evaluated or discussed. This study aimed to determine the efficacy, safety, and diagnostic yield of a percutaneous ultrasound‐guided biopsy of peritoneal masses.

## 2 MATERIALS AND METHODS

### 2.1 Study design

The study protocol was evaluated and approved by the relevant Pamukkale University review board (E‐60116787‐020‐279,062). We retrospectively reviewed and recorded the electronic medical records of patients who underwent PCNB for peritoneal lesions in the Department of Interventional Radiology at our institution between January 1, 2016 and October 31, 2023. Out of a total of 189 patients, those who underwent CT guidance, employed the coaxial method, lacked available CT images, and those for whom noncontrast CT images were not accessible were excluded from the study. The 129 ultrasound‐guided PCNB procedures included those with sufficient data that could be accessed from electronic records.

### 2.2 Technique

**FIGURE 1:**
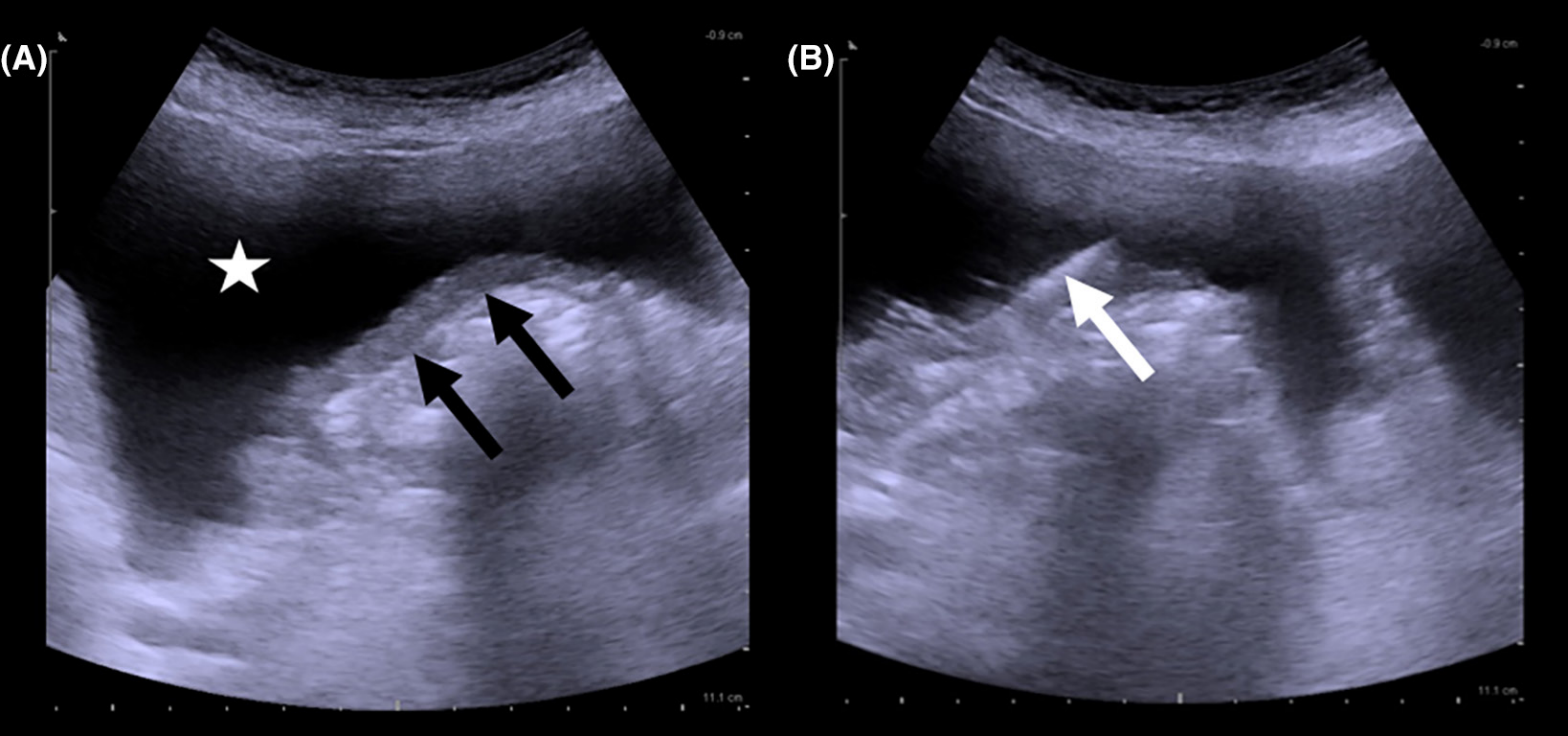
A 76‐year‐old woman presented with no prior history of cancer who was hospitalized with abdominal distension and an omental mass. Ultrasound images (A, B) show a thickened omentum (black arrows) and an 18‐gage core biopsy needle (white arrow) with ascites (asterisk). Her pathological diagnosis was high‐grade ovarian serous adenocarcinoma metastasis.

Informed consent was obtained in written format from all patients prior to the procedure. Patients were screened for coagulopathy prior to biopsy to ensure a serum platelet count of ≥50,000 μL or greater and an international normalized ratio of 1.5 or less. All procedures were performed by two radiologists in the Interventional Radiology Suite. The imaging modality for guidance was ultrasound (US), and the patients underwent PCNB using an auto biopsy gun with 18‐gage cutting biopsy needles (Bard Biopsy, Bard Medical Division, Tempe, AZ) under local anesthesia (Figure 1). Two blood samples were collected from each patient. Generally, the entry is performed twice using the same needle. The samples were fixed in formalin and sent for pathological examination. After the procedure, the patients were instructed to stay in bed for at least 6 h, and their vital signs and peritoneal symptoms were monitored. Before the patient was discharged, a complete blood count and an US control were performed.

### 2.3 Lesion imaging characteristics and localization

**FIGURE 2:**
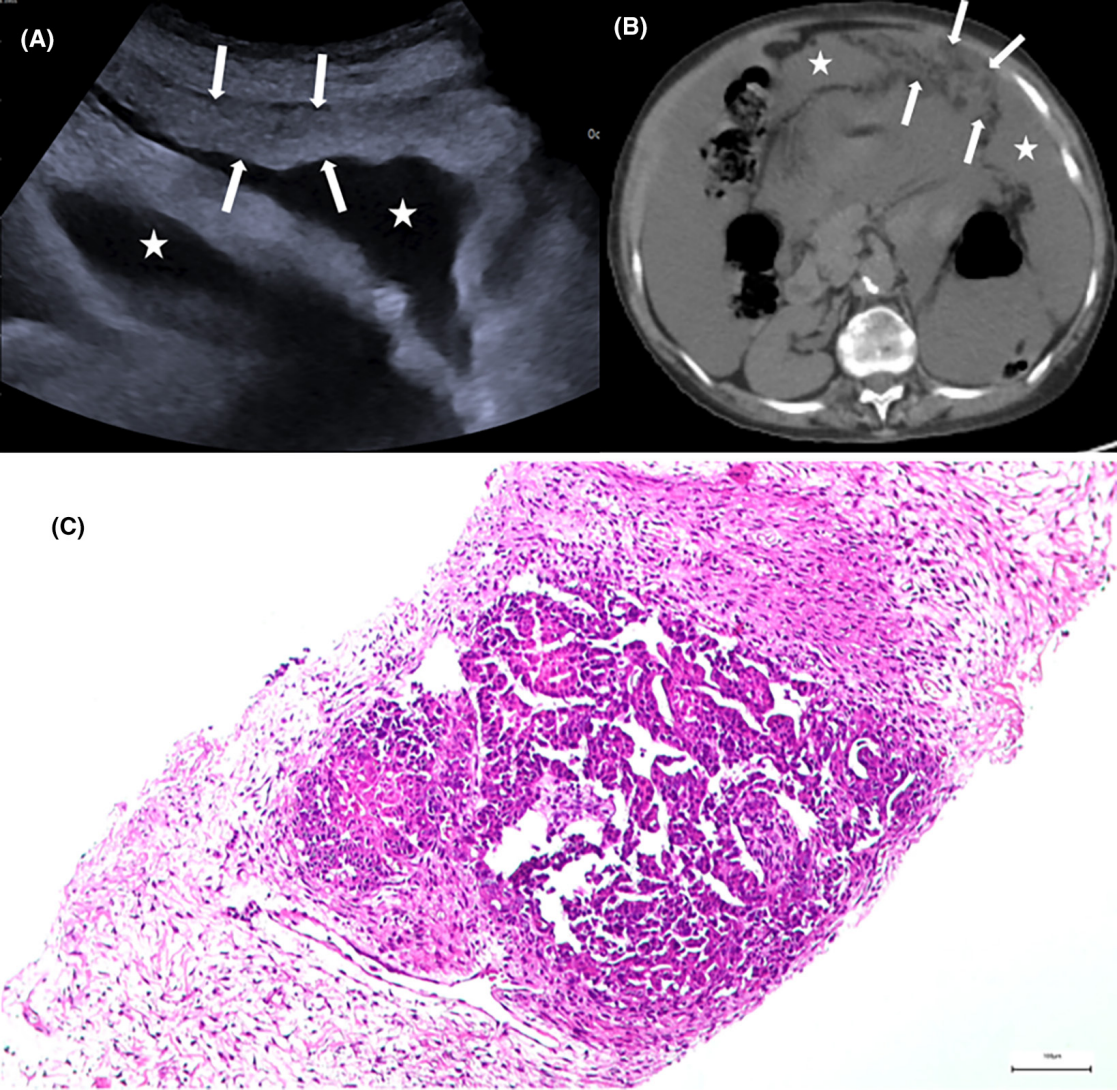
A 62‐year‐old woman presented with abdominal distension due to metastatic serous ovarian carcinoma. (A) Ultrasonography shows diffuse thickening of the peritoneum (arrows) and ascites (asterisks) in the left lower quadrant. (B) Unenhanced computed tomography image reveals large‐volume ascites (asterisks) with heterogeneous soft tissue attenuation (arrows) along the left anterior parietal peritoneum. (C) Histopathological examination of the tumor reveals a papillary growth pattern with rare fibrovascular cores and slit‐like cavities within the fibrous stroma. Tumor cells exhibit moderate nuclear pleomorphism and hyperchromasia (H&E, ×200 magnification).

The anteroposterior (AP) diameter and skin‐to‐lesion measure were evaluated on axial CT images. The presence of ascites was evaluated using CT before PCNB was performed. Peritoneal lesions were subdivided into two groups depending on the imaging characteristics of the omental abnormality at the time of biopsy. Nodular lesions were defined as solitary or multiple discrete round‐shaped omental lesions, while diffuse lesions were defined as diffuse peritoneal thickening and confluent nodular lesions (Figure 2). The abdomen was divided into four quadrants, with vertical and horizontal dividing lines at the level of the umbilicus. The target lesion quadrant was recorded on a worksheet. The Hounsfield unit value of the lesion on CT was evaluated on noncontrast CT.

### 2.4 Complications

Complications were classified according to the Society of Interventional Radiology guidelines for needle biopsy.^4^ Major complications were defined as events leading to substantial morbidity and disability that increased the level of care, required further therapy, or prolonged hospitalization. Major bleeding, infection, adjacent organ injury, and death were defined as major complications. All other complications, such as pain, skin numbness, skin hematoma, transient hypotension, vomiting, lidocaine toxicity, and cough, were considered minor.

### 2.5 Statistical analysis

The technical success rate, diagnostic yield, and the complications were evaluated in this study. Technical success was defined as completion of the planned biopsy, with a possible pathological evaluation. The diagnostic yield of the procedure was determined based on the diagnostic biopsy results using chi‐square tests in relation to sex, pre‐existing ascites, history of malignant disease, localization of the target lesion, lesion imaging characteristics, guiding method, and coaxial technique. Age, AP diameter, distance of the lesion from the skin, and the Hounsfield unit value of the target lesions on CT were evaluated using the Mann–Whitney U test in relation to diagnostic yield. Logistic regression analysis was employed to discern the factors influencing the adequacy of pathological results. During the univariate analysis, the impact of each variable was assessed in isolation. In the multivariate model, variables deemed potentially influential were incorporated for a comprehensive evaluation.

## 3 RESULTS

One hundred and twenty‐nine patients who underwent an ultrasound‐guided percutaneous peritoneal lesion biopsy were included in this study. Demographic information and CT characteristics of the peritoneal lesions are shown in Table 1. Specimens were obtained for pathological diagnosis in all cases, with a technical success rate of 100%. An adequate sample size for the pathological evaluation was obtained from 116 patients (89.9%). Malignant biopsy results were obtained in 104 (89.7%) patients, and histopathological results showed benign disease in 12 (10.3%) patients with enough biopsy tissue. The pathological results for all patients with sufficient biopsy tissue are presented in Table 2. A pathological diagnosis was not obtained in 13 patients. Among these 13 patients, two patients were diagnosed with normal small intestinal mucosa, one with fat and muscular tissue, four with suspected malignancy, and seven with nondiagnostic specimens based on pathological results. Biopsy was performed in 48 patients with a known history of cancer. Among these 48 patients with a known cancer history, 30 patients' results were consistent with primary cancer, six patients were diagnosed with benign pathologies, and five patients were diagnosed with an additional malignancy. Seven patients with a known cancer history were not diagnosed due to insufficient samples. A new malignancy was detected in 70 patients without a known cancer history.

**TABLE 1:** Demographic and clinical variables in patients undergoing percutaneous ultrasound‐guided peritoneal biopsy.

	Pathological Result		
	Negative (*n* = 13)	Positive (*n* = 116)	*p*‐Value
Gender^a^			
Male	4 (30.7)	39 (33.6)	0.837^c^
Woman	9 (63.3)	77 (66.4)	
Age^b^	62 [44.0–82.0]	65.3 [30.0–85.0]	0.763^c^
History of malignancy, yes ^a^	9 (69.2)	40 (34.5)	**0.017** ^c^
Presence of ascites, yes ^a^	10 (76.9)	84 (72.4)	0.510^c^
Skin‐to‐lesion measure (mm)^b^	35.2 [19–58]	31.2 [10–60]	0.324^c^
Anteroposterior diameter (mm)^b^	18.5 [9–35]	31.3 [5–170]	**0.001** ^c^
Density of target lesion (HU)^b^	[−3–39]	[−25–88]	0.334^c^
Peritoneal lesion type^a^			
Nodular lesion	2 (15.4)	27 (23.3)	0.404^c^
Diffuse lesion	11 (84.6)	89 (76.7)	
Lesion quadrant localization^a^			
Right upper	3 (23.1)	31 (26.7)	0.278^c^
Left upper	5 (38.5)	19 (16.4)	
Right lower	3 (23.1)	41 (35.3)	
Left lower	2 (15.4)	25 (21.6)	

^a^ *n*(%).

^b^ Median [Min.‐Max.].

^c^ Mann–Whitney *U* test.

*Note*: Bold Font Statistically significant results.

**TABLE 2:** Histopathological results.

Pathology results	Number	Percentage (%)
Ovarian cancer metastasis	57	49.13
Pancreas cancer metastasis	10	8.62
Colon cancer metastasis	8	6.89
Endometrial cancer metastasis	5	4.31
Malign epithelial neoplasm metastasis	4	3.44
Lung cancer metastasis	4	3.44
Other malignancies^a^	16	13.79
Granulomatous inflammation	8	6.89
Pseudomyxoma peritonei	2	1.72
Cyst hydatid	1	0.86
Solitary fibrous tumor	1	0.86

^a^ Other malignancies: genitourinary malignancies metastasis, lymphoma, breast carcinoma metastasis, primary peritoneal mesothelioma, liposarcoma, gallbladder carcinoma metastasis, malign melanoma metastasis, appendix mucinous neoplasm metastasis.

When examining patient and lesion characteristics based on positive results, it was determined that a known history of cancer and AP diameter of the target lesion significantly affected the diagnostic yield of PCNB. However, other factors were not statistically significant for the diagnostic yield of PCNB (Tables 1, 3). Two major complications and 11 minor complications were reported in the first 24 h following the procedure. Major complications included intraperitoneal bleeding (two patients, 1.5%), minor complications included pain (six patients, 6.2%), a skin hematoma (two patients, 1.5%), and vasovagal reflex (one patient, 0.75%). Our two major complications involved instances of bleeding, which were noted in patients with peritoneal drainage catheters. Upon the observation of ascitic fluid exhibiting a red coloration, we closely monitored the patient, and within hours, the color of the ascitic fluid returned to a lighter shade.

**TABLE 3:** Logistic regression analysis on factors that may influence sufficient pathological results.

Dependent variable: Sufficient biopsy	Univariate				Multivarite			
	*p*	O.R	95% C.I. for O.R.		*p*	O.R	95% C.I. for O.R.	
			Lower	Upper			Lower	Upper
Age	0.266	0.956	0.882	1.035				
Gender	0.177	0.282	0.045	1.768				
A known history of cancer	**0.021**	4.275	1.239	14.750	**0.032**	4.105	1.128	14.943
Anteroposterior diameter	**0.007**	1.109	1.029	1.194	**0.013**	1.094	1.019	1.174
Skin‐to‐lesion measure	0.347	0.971	0.912	1.066				
Peritoneal lesion Type	0.261	2.483	0.291	21.209				
CT value of target lesion (HU)	0.248	1.024	0.984	1.066				
Presence of ascites	0.729	0.788	0.204	3.047				
Lesion quadrant localization	0.514	1.191	0.705	2.014				

Abbreviations: CI, Confidence interval; CT, Computed tomography; HU, Haunsfield unit; OR, Odds ratio; PCNB, percutaneous core needle biopsy,

*Note*: Bold Font Statistically significant results.

## 4 DISCUSSION

The peritoneum can be involved in metastatic diseases, primary tumors, infections, other inflammatory conditions, and hemorrhage.^5^ Pathological diagnosis is necessary because radiological diagnosis is usually insufficient. This study confirms that ultrasound‐guided PCNB is a feasible method with a high technical success rate which has also been observed in previous studies.^3^, ^5^, ^6^, ^7^ The diagnostic yields reported in previous studies were also consistent with those reported in the present study.^3^, ^5^, ^6^, ^7^, ^8^ In addition, the most common pathological finding in this study was ovarian cancer, which is consistent with the findings of other studies.^3^, ^7^, ^9^

Most PCNB procedures are performed under US or CT guidance. Equipment availability, location of the target lesion, adequacy of lesion visualization, patient comorbidities, and cost affect the choice of modality.^10^ US is increasingly used as the first choice for image guidance. Our reasons for selecting the US include its accessibility, real‐time visualization capabilities, absence of ionizing radiation, and shorter procedure duration. We were able to observe lesions not visible on the initial US examination by re‐examining according to anatomical markers from cross‐sectional images. US was sufficient for performing peritoneal biopsies in many cases.

This study demonstrated that only a known history of cancer was associated with the diagnostic yield, which was an unexpected result. The high rate of nondiagnostic samples with a history of malignancy may be due to the fact that samples are taken from more difficult patients with the expectation of metastasis, or it may be due to the difficulty in making pathological decisions in patients with a known primary malignancy. Statistical analysis indicated that the AP diameter of the target lesion was significant for diagnostic yield. In the study of Sugawara et al. evaluating the diagnostic yield of PCNB for target lesion size, the diagnostic yield for small lesions was lower than long lesions, consistent with the present study.^3^ However, two prior study reported the diagnostic yield of the procedure was not influenced by the target lesion size. In a previous studies evaluating the diagnostic yield of PCNB for target lesion size, the diagnostic yield for small lesions was lower than long.^5^, ^6^

PCNB of peritoneal lesions is a safe and well‐tolerated procedure, regardless of the presence of ascites. Major complications, such as abscess, rectus sheath hematoma, mesenteric hematoma, and colon perforation have been reported as complications of PCNB.^6^, ^11^ Two major complications were observed in the current study. Nevertheless, these bleeding complications were successfully managed solely through monitoring, without necessitating any additional procedures or blood transfusions. Ascites is relatively contraindicated owing to the risk of bleeding in many interventional procedures, such as liver biopsy and percutaneous biliary drainage. Since most cases of peritonitis carcinomatosis are associated with ascites, most peritoneal biopsies (69.7%) in our study revealed ascites. Despite this, we did not observe the need for additional procedures for our two major complications. It has also been reported that the presence of ascites does not increase the incidence of complications in peritoneal biopsies.^3^ Despite unexpected pathological results, such as normal intestinal mucosa or hydatid cysts, no complications occurred.

The current study has some limitations. First, it was retrospective, with an inherent selection bias and the risk of missing data. Second, as not all CT scans included contrast‐enhanced images, therefore, we used only noncontrast images. Another limitation is that the short clinical follow‐up period in some cases may have influenced the presence of long‐term complications, such as needle tract seeding. Theoretically, the coaxial technique allowed us to collect more than one sample with a single entry and to simultaneously collect ascitic fluid for cytological examination from the same needle. However, we did not perform a statistical analysis in terms of diagnostic yield and complications because the coaxial technique was used in a small number of cases in our study.

## 5 CONCLUSIONS

Ultrasound‐guided PCNB is an effective, safe, and well‐tolerated method for evaluating peritoneal lesions. Most biopsies can be performed under US guidance. We found that a known history of cancer and the AP diameter of the target lesion affect the diagnostic yield of PCNB.

## AUTHOR CONTRIBUTIONS

**Muhammet Arslan:** Conceptualization (equal); data curation (equal); formal analysis (equal); investigation (equal); methodology (equal); project administration (equal); supervision (equal); writing – original draft (equal); writing – review and editing (equal). **Halil Serdar Aslan:** Conceptualization (equal); data curation (equal); resources (equal). **Ayse Ruksan Utebey:** Methodology (equal); writing – original draft (equal). **Sercan Vurgun:** Data curation (equal); methodology (equal). **Erdem Comut:** Data curation (equal); formal analysis (equal); resources (equal). **Serkan Degirmencioglu:** Data curation (equal); methodology (equal). **Derya Kılıc:** Data curation (equal); investigation (equal); writing – original draft (equal).

## FUNDING INFORMATION

Open access funding is provided by Read & Publish agreement which was signed between TUBİTAK ULAKBİM and Wiley Publishing House in February 2023. The authors did not receive support from any organization for the submitted work.

## CONFLICT OF INTEREST STATEMENT

The authors disclosed no conflicts of interest.

## ETHICS STATEMENT

The study protocol was evaluated and approved by the relevant Pamukkale University review board (E‐60116787‐020‐279,062).

## CONSENT

Informed consent was obtained using a written form from all patients prior to the procedure.

## Data Availability

The data that support the findings of this study are not openly available due to reasons of sensitivity and are available from the corresponding author upon reasonable request.
